# Velpeau on Fractures

**Published:** 1846-11

**Authors:** 


*Velpeau on Fractures.*—The following extracts are from a report of cases treated by M. Velpeau, surgeon to the Hospital "La Charite," from Sept. 1845 to Sept. 1836; communicated for the Boston Medical and Surgical Journal, by a Paris correspondent.

"The fractures of the clavicle, less numerous than ordinarily, have been only four. They have proved these three often-repeated propositions: first, that, contrary to the general opinion, the patients can carry the hand to the head when they have a fractured clavicle; secondly, that the consolidation of the bone demands only from fifteen to twenty-five days, and not six weeks or two months; thirdly, that with all the bandages imaginable, we cannot prevent fracture of the two internal and oblique thirds from leaving a deformity. All the treatment consists in preventing any movement for about twenty days."

"There were five fractures of the neck and two of the body of the femur. Two of these were compound fractures. The fractures of the neck have all been treated without extension; the limb has been simply placed upon the double inclined plane, and the patients were permitted to walk in proper season. All have been discharged, not exactly cured, but in a satisfactory condition, excepting the lameness which is unavoidable. The reasons why continued extension has not been used, are, first, because it does not prevent lameness; second, the shortening is not diminished, and the employment of extension infallibly produces sloughs, œdema and considerable stiffness. Moreover, and this is an important consideration, force obliges patients to keep their beds for two or three months, and as fractures of the neck of the femur occur mostly in persons advanced in life, they soon fall into an adynamic state and sink. On the contrary, with the simple treatment of Velpeau, they are as well at the end of six weeks as they could be at the expiration of six months under the use of bandages and continued extension. It is necessary to know that raising up the patient does not prevent the union; as they walk with crutches, the pain warns them not to place the affected limb on the ground, and the suspension which it occasions, produces a sort of extension by the weight of the limb itself. Ought all patients to be put under this treatment? No. If the subject is young, in order to obtain a good consolidation, it is necessary to employ suitable dressings.

There have been two fractures of the patella. As for the treatment, the parts are brought together moderately, the patient remains in bed two or three weeks, and then gets up, and fifteen days afterwards his bandage is removed."

